# *N*-Acetylcysteine Alleviates Impaired Muscular Function Resulting from Sphingosine Phosphate Lyase Functional Deficiency-Induced Sphingoid Base and Ceramide Accumulation in *Caenorhabditis elegans*

**DOI:** 10.3390/nu16111623

**Published:** 2024-05-26

**Authors:** Min Liu, Yunfei You, Huaiyi Zhu, Yu Chen, Zhenying Hu, Jingjing Duan

**Affiliations:** Jiangxi Province Key Laboratory of Aging and Disease, Human Aging Research Institute (HARI), School of Life Science, Nanchang University, Nanchang 330031, China

**Keywords:** sphingosine-1-phosphate lyase, muscle, *Caenorhabditis elegans*, ceramides, sphingolipids

## Abstract

Sphingosine-1-phosphate lyase (SPL) resides at the endpoint of the sphingolipid metabolic pathway, catalyzing the irreversible breakdown of sphingosine-1-phosphate. Depletion of SPL precipitates compromised muscle morphology and function; nevertheless, the precise mechanistic underpinnings remain elusive. Here, we elucidate a model of SPL functional deficiency in *Caenorhabditis elegans* using *spl-1* RNA interference. Within these SPL-deficient nematodes, we observed diminished motility and perturbed muscle fiber organization, correlated with the accumulation of sphingoid bases, their phosphorylated forms, and ceramides (collectively referred to as the “sphingolipid rheostat”). The disturbance in mitochondrial morphology was also notable, as SPL functional loss resulted in heightened levels of reactive oxygen species. Remarkably, the administration of the antioxidant *N*-acetylcysteine (NAC) ameliorates locomotor impairment and rectifies muscle fiber disarray, underscoring its therapeutic promise for ceramide-accumulation-related muscle disorders. Our findings emphasize the pivotal role of SPL in preserving muscle integrity and advocate for exploring antioxidant interventions, such as NAC supplementation, as prospective therapeutic strategies for addressing muscle function decline associated with sphingolipid/ceramide metabolism disruption.

## 1. Introduction

**Figure 1:**
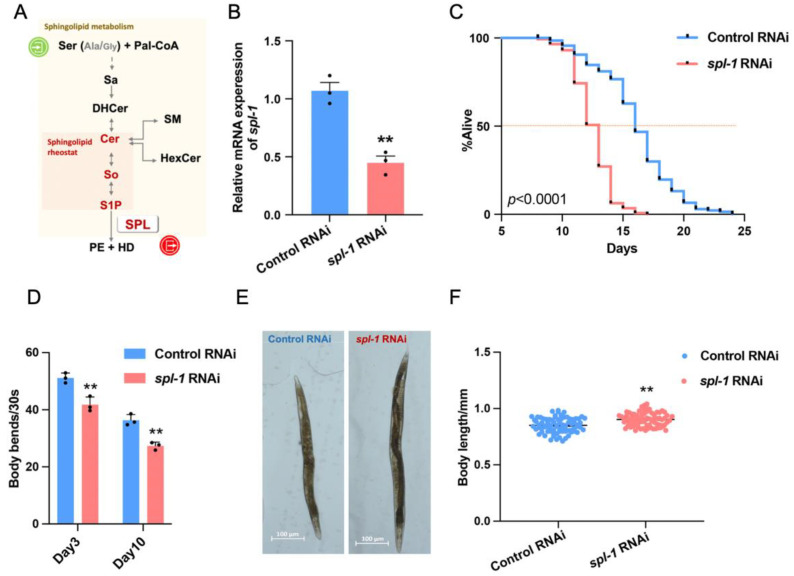
**The *spl-1* RNAi reduces lifespan and induces impaired locomotion in *Caenorhabditis elegans.*** (**A**) Sphingosine-1-phosphate lyase (SPL) is sphingosine-1-phosphate (S1P) degrading enzyme, which resides at the exit of the sphingolipid metabolic network, and the dynamic balance between ceramides (Cer) and S1P is called a “sphingolipid rheostat”. (**B**) The RNA interference efficiency of *spl-1* was assessed using RT-qPCR, reaching 50% and revealing a significant difference compared with the Control RNAi, as analyzed by two-tailed Student’s *t* test (t = 6.88, ** *p* < 0.01). (**C**) The survival curves of Control RNAi (*n* = 137) and *spl-1* RNAi (*n* = 144)-treated *C. elegans* were estimated. Days were counted from the L4 stage as day 0 for all animals (abscissa). (**D**) The body bands at day 3 and day 10 were assessed; *n* = 3 for each group; each independent repeat experiment detected 10 worms. (**E**) Images of Control and *spl-1* RNAi-treated nematodes at the day 3 stage captured using a Zeiss optical microscope. (**F**) The body lengths of nematodes at the day 3 stage were analyzed; *n* = 80 for each group. Results are presented as mean values. The *p* values are relative to Control RNAi-treated animals analyzed by a two-tailed Student’s *t* test (** *p* < 0.01).

Sphingosine phosphate lyase (SPL) resides at the terminal of the sphingolipid metabolic network, catalyzing the breakdown of sphingosine-1-phosphate (S1P) to produce ethanolamine phosphate and hexadecenal [1]. This enzyme is pivotal for maintaining sphingolipid homeostasis and regulating sphingolipid signaling transduction [2]. SPL deficiency disrupts S1P metabolism and its precursors: sphingoid bases and ceramides, which act as key regulators of cellular fate, termed the “sphingolipid rheostat” [3] (Figure 1A). The accumulation of sphingolipids due to SPL deficiency disrupts the cellular signaling pathways involved in proliferation, survival, and migration, leading to various physiological and pathological consequences [4,5,6]. For instance, SPL defects elevate S1P levels in the bloodstream, affecting vascular permeability and immune cell migration [7,8]. Additionally, SPL deficiency is associated with nervous and immune system abnormalities, as well as muscle fiber disorganization [9,10,11].

SPL deficiency significantly influences larval survival in fruit flies and nematodes, as well as muscle development in adult flies [12,13]. SPL-deficient mice exhibit bone marrow cell proliferation and varying degrees of lesions in the heart, lungs, bones, and urinary tract [14]. Targeted knockout of the SPL-encoding *Sgpl1* gene leads to a significant accumulation of ceramides, S1P, and sphingomyelins in mouse serum, resulting in smaller body size and severe motor function deterioration [15]. However, the mechanisms underlying SPL-mediated muscle function decline remain unclear. Sphingolipid metabolic homeostasis, particularly, the balance of each member of the “sphingolipid rheostat”, plays a crucial role in skeletal muscle function [16]. Recent studies have highlighted the significance of ceramides in skeletal muscle integrity [17]. Skeletal muscle biopsies from elderly mice and humans have shown reduced mRNA expression levels of ceramide synthase (CerS) 1 and CerS5 compared with younger counterparts [18]. Genetic knockout of CerS1 and CerS5 in mice leads to aberrant muscle fiber structure and impaired function, underscoring their importance in skeletal muscle physiology [19]. Moreover, the analysis of the transcriptional abundance of enzymes involved in the *de novo* sphingolipid biosynthesis pathways in young and elderly individuals revealed notable upregulation of SPTLC1 (serine palmitoyltransferase long-chain base subunit 1), KDSR (3-ketodihydro-sphingosine reductase), CERS2 (ceramide synthase 2), and CERS5, with downregulation observed in CERS1 [20]. Primary myoblasts from elderly individuals and those with inclusion-body myositis demonstrate elevated concentrations of sphingoid bases and ceramides [21]. Furthermore, aging myoblasts display reduced oxygen consumption and cellular respiratory efficiency. The inhibition of *de novo* sphingolipid synthesis using the serine palmitoyl transferase inhibitor myriocin enhances cellular oxygen consumption, suggesting a potential strategy to ameliorate muscle health by preventing excessive sphingolipid production [20].

A readily accessible, operable, and reproducible model of disrupted sphingolipid metabolism, coupled with a motor dysfunction model, can prove invaluable for investigating interventions targeting muscle function impairments arising from sphingolipid metabolism disorders [22]. The well-defined genetic background and lifecycle of *Caenorhabditis elegans* offer an ideal platform for such investigations. Therefore, in this study, we employed *C. elegans* to target the key enzyme involved in the sphingolipid metabolism exit, SPL, and employed sphingolipidomics alongside motor phenotype analysis to delve into the mechanisms underpinning motor dysfunction resulting from sphingolipid metabolism disorders, thereby initiating exploration into potential safe and effective intervention strategies.

## 2. Materials and Methods

### 2.1. Culture of Nematode Strains

The Nematode Bristol strain (N2, wildtype) and reporter strains SJ4103 [*myo-3::GFP(mit)*] and RW1596 [*myo3p::GFP::myo-3+rol-6(su1006)*] were obtained from the Caenorhabditis Genetics Center. The nematodes were cultured on nematode growth medium (NGM) agar plates supplemented with *Escherichia coli* strain OP50 as a food source.

### 2.2. RNAi Experiments

RNAi constructs targeting *spl-1* were sourced from the Open Biosystems ORF-RNAi library and subjected to sequence verification prior to experimentation. Synchronized L4-stage *C. elegans* nematodes were employed. The RNAi-treated strains were fed with *E. coli* HT115 bacteria containing either an L4440 empty vector as the Control RNAi or a vector expressing double-stranded RNAi targeting *spl-1*. The induction of double-stranded RNA expression was achieved by treating with 1 mM isopropyl 1-thio-*β*-D-galactopyranoside.

### 2.3. Lifespan Assay

A lifespan assay was carried out at 20 °C. Briefly, nematodes at the reproductive stage were collected to synchronize, and eggs were incubated in M9 buffer at room temperature overnight. Subsequently, L1-stage nematodes were transferred to nematode growth medium (NGM) plates seeded with *E. coli* OP50. Upon reaching the L4 stage, worms were transferred to NGM plates seeded with *E. coli* HT115. Throughout adulthood, the worms were transferred daily to fresh NGM plates with various treatment groups until the death of the last worm, to prevent interference from progeny and facilitate counting. An animal was deemed dead if it displayed no spontaneous movement and did not respond to prodding with a platinum wire. The synchronized eggs that hatched were designated as day 0.

### 2.4. Pharyngeal Pumping Rate Assay

After the L4 stage, the worms treated with HT115-seeded NGM plates for 3 and 10 days were collected to assess pharyngeal pumping by observing the number of pharyngeal contractions during a 30 s interval. The pharyngeal pump rate in the treated group and the control group was tested in three parallel experiments, each parallel with ten nematodes.

### 2.5. Body Movement Assay

After reaching the L4 stage, the worms were treated with HT115-seeded NGM plates for 3 and 10 days. Subsequently, they were collected to assess body movement by observing the number of pharyngeal contractions during a 30 s interval. The body movement in both the treated and control groups was tested in three parallel experiments, each with ten nematodes.

### 2.6. Body Length Assay

The synchronized worms were cultured on NGM plates seeded with OP50 bacteria until reaching adulthood. Subsequently, the worms were treated with HT115-seeded NGM plates for 3 days. Their body lengths were measured at various stages using an optical microscope (Zeiss, Primostar 3, Oberkochen, Germany) equipped with Zen software (ZEN Blue 3.4).

### 2.7. Myofiber and Mitochondria Morphology Measurements 

SJ4103 and RW1596 nematodes were cultured on NGM plates seeded with HT115 for 3 days, followed by three washes with M9 buffer. Subsequently, the nematodes were transferred into fluorinated oil and placed onto slides coated with 2% agarose (approximately 1 mm thick) to ensure their viability. Imaging of the nematodes was then performed using confocal laser scanning microscopy (Zeiss, LSM800, Oberkochen, Germany). Phalloidin staining was also conducted to observe actin filaments in the muscle sarcomeres. We conducted three independent experiments and integrated the analysis of the image results, focusing on specific regions.

### 2.8. ROS Measurement 

Synchronized nematodes at day 3 of adulthood were washed with M9 buffer (22 mmol/L KH_2_PO_4_, 22 mmol/L Na_2_HPO_4_, 85 mmol/L NaCl, and 1 mmol/L MgSO_4_) and then transferred to 2 mL of M9 buffer containing 10 µM dichlorofluorescein diacetate (DCFH-DA, HY-D0940; MedChemExpress, Monmouth Junction, NJ, USA) for total ROS or 10 µM MitoSOX Red (40778ES50, YEASEN, CHN) for mitochondria ROS, and MitoSOX Red indicator for the specific detection of mitochondrial superoxide in live cells [23]. After incubation for 30 min at 20 °C in the dark, the nematodes were rinsed with M9 buffer and anesthetized with 10 mM L-imidazole hydrochloride for 5 min. Subsequently, the worms were imaged using a confocal laser scanning microscope.

### 2.9. N-Acetylcysteine Treatment

*N*-acetylcysteine (NAC) was dissolved in sterile water to prepare a 1 M stock solution, which was then filtered. We pre-seeded *spl-1* interference bacteria on nematode culture plates containing 10 mM NAC-IPTG. After culturing the L4-stage N2 nematodes in this medium for 3 days, we conducted assays to measure the ROS levels, as well as the muscle fiber and mitochondrial morphology, in the nematodes.

### 2.10. Lipid Extraction

For both sets of targeted sphingolipids (including id17:0, id17:1, id17:0-S1P, id17:1-S1P, Cer, SM, and HexCer), approximately 5000 nematodes from each experimental group were used for lipid extraction. Initially, the worm samples were freeze-dried overnight and then homogenized with 1 mL of H_2_O. Then, 900 μL of each homogenized sample was transferred to new glass tubes. The extraction process required adding 1 mL of methanol and 0.5 mL of either dichloromethane (for sphingoid bases analysis) or chloroform (for Cer, SM, and HexCer analysis), followed by vortexing for 1 min. Each tube was then incubated at 48 °C for 24 h after mixing with internal standard (25 μmol #LM-6005, Avanti Polar Lipids, Alabaster, AL, USA). After incubation, 150 µL of 1 M KOH in methanol was added, briefly sonicated, and then shaken at 37 °C for 2 h. The solutions were neutralized with glacial acetic acid (8 µL for sphingoid bases and 6 µL for Cer, SM, and HexCer). After centrifugation, the solutions were carefully collected, and any residue was re-extracted using the appropriate solvent. Following evaporation with a speed vacuum to avoid overheating, the residues were dissolved in LC solvents for LC-MS/MS analysis. The remaining 100 μL from each sample was used to quantify proteins to normalize the sphingolipid content as pmol/mg. This streamlined approach efficiently combines the extraction steps for both sets of lipids, ensuring a concise and coherent protocol.

### 2.11. Sphingolipid Analysis

The sphingolipid analysis followed the protocol adopted from the LIPID MAPS protocols (URL: www.lipidmaps.org (accessed on 16 January 2024)) and our published method for sphingolipidomic analysis of *C. elegans* [6], with slight modifications, utilizing an ultra-high-performance liquid chromatography (UPLC) system (Shimadzu, Kyoto, Japan) coupled with a Triple Quad™ 5500^+^ QTRAP^TM^ (AB Sciex, Vaughan, ON, Canada). The UPLC system comprised a binary pump system (LC-30AD), a degasser (DGU-20A5), a temperature-controlled autosampler (SIL-30AC), a column oven (CTO-20AC), and a control unit (CBM-20A). The mass spectrometer was operated in positive electrospray mode coupled with multiple reaction monitoring (MRM) transitions of the mass-to-charge ratio (*m/z*), as detailed in Supplementary Table S2. The ion source (ESI) voltage was set at 4.5 kV, and the ion source temperature was 400 °C. The ion source gas 1/2 and curtain gas flows were 60 psi and 40 psi, respectively. The dwell time and the inter-channel delay were set to 30 and 5 ms. The MS system was controlled by Analyst 1.7.3 software (Applied Biosystems, Waltham, MA, USA). For chromatographic separation, the LC conditions are summarized in Table S3 (Method #1 for sphingoid bases and Method #2 for Cer, SM, and HexCer). For each LC analysis, mixture of standards (C17-sphinganine, C17-sphingosine, C17-sphingosine-1-phosphate, C17-sphinganine-1-phosphate, C12-ceramide, C12-ceramide-1-phosphate, C12-SM, C12-glucosylceramide, C12-lactosyl (β)-ceramide (Avanti Ploar Lipids, Alabaster, AL, USA)) were analyzed at the beginning, middle, and end of the run. In addition, the LC solvent as a blank was analyzed at varying intervals throughout the run to assess for possible carryover. If carryover or shifts in the LC retention times for any of the analytes or standards were noticed, the column was cleaned before resuming the run. The data analysis including peak smoothing and integration of areas under the curves for each ion was performed by SCIX OS (Version.3.0.0.3339, Applied Biosystems, Waltham, MA, USA). The semi-quantitation of individual metabolites was normalized with the respective internal standards and calculated with the following formula: Analyte Conc. (nmol/L) in worm sample = Area (analyte MRM peak) × Standard Conc. (nmol/L) / Area (Standard MRM peak). In the heatmap, each group was standardized against the N2 (wildtype) group’s average to show the variations in individual lipid. 

### 2.12. Reverse-Transcription PCR

The total RNA of the nematodes was extracted using the RNAiso Plus reagent (Takara Bio, Tokyo, Japan). The PCR products were amplified and quantified using the SYBR Green Real-Time PCR Supermix (Mei5 Biotechnology, Beijing, China). The transcriptional expression levels of the target genes were normalized to *act-1*, which served as the internal control. The primers used for qRT-PCR are listed in Supplementary Table S1.

### 2.13. Statistical Analysis 

The lifespan curves were compared using the Kaplan–Meier survival method and analyzed by the log-rank test. All other data are presented as the mean ± SD unless specifically indicated. The statistical analyses included two-tailed Student’s *t* test or one-way ANOVA, * *p* < 0.05; ** *p* < 0.01; *** *p* < 0.05. All the figures were generated using GraphPad Prism 10 (GraphPad Software, La Jolla, CA, USA).

## 3. Results

### 3.1. Spl-1 RNA Interference Reduces Lifespan and Impairs Locomotion in Caenorhabditis elegans

To investigate the impact of SPL deficiency on *C. elegans*, an SPL knockdown model was established using HT115 bacteria carrying dsRNA targeting the *spl-1* gene (Figure 1A). And, compared with the Control RNAi (L4440), there was a significant reduction in *spl-1* mRNA level in N2 worms exposed to RNA interference bacteria (Figure 1B). The lifespan analysis revealed a significant reduction in both average and maximum lifespans in the *spl-1* RNAi nematodes (Figure 1C). Interference with *spl-1* gene expression significantly decreased body bends in 30 s, reflecting impaired locomotion (Figure 1D). Additionally, reduced egg laying was observed upon SPL gene knockdown (Supplementary Figure S1A), along with enlarged body sizes (Figure 1E,F). Furthermore, the loss of SPL function did not significantly affect the pharyngeal pumping ability of *C. elegans*, regardless of their developmental stage (Supplementary Figure S1B), indicating that the diverse effects observed upon SPL gene knockdown were not due to insufficient food intake induced by pharyngeal pumping.

### 3.2. SPL Loss-of-Function Leads to Accumulations of Sphingoid Bases and Ceramides in C. elegans

**Figure 2:**
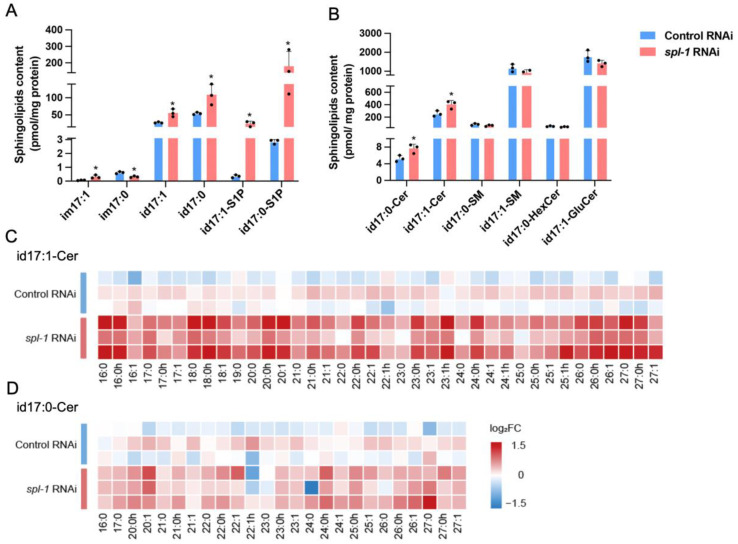
**Sphingoid bases and ceramides accumulate in SPL loss-of-function *C. elegans***. (**A**) Content analysis of sphingoid bases (typical sphingosine id17:1, sphinganine id17:0; atypical 1-deoxysphingosine im17:1, and 1-deoxysphinganine im17:0), and their 1-phosphate forms (so-called sphingosine-1-phosphate, S1P) id17:1-S1P and id17:0-S1P. (**B**) The contents of total dihydroceramides (id17:0-Cer), ceramides (id17:1-Cer), dihydrosphingomyelin (id17:0-SM), sphingomyelin (id17:1-SM), dihydrohexosylceramide (id17:0-HexCer), and hexosylceramide (id17:1-HexCer). (**C**) Heatmaps display the relative changes in the log2 fold change of the C16~C27 *N*-acyl chain lengths of dihydroceramides (id17:0-Cer) and (**D**) ceramides (id17:0-Cer) in nematodes, generated using the R pheatmap package. The *p* values, compared with the Control RNAi-treated worms, were determined by a two-tailed Student’s *t*-test (* *p* < 0.05).

To assess the impact of *spl-1* knockdown on nematode sphingolipid metabolism, we conducted sphingolipidomics to analyze the molecular composition and content of sphingoid bases (sphingosine id17:1, sphinganine id17:0), their phosphate forms (also called sphingosine-1-phosphate, S1P), ceramides (Cer), hexosylceramides (HexCer), and sphingomyelins (SM) with C16~C27 *N*-acyl chain length and, in addition, atypical 1-deoxysphingosine im17:1 and 1-deoxysphinganine im17:0 in nematodes. The analysis reveals a disturbed sphingolipid metabolism caused by *spl-1* RNAi. It was evident that there was a significant accumulation of sphingoid bases, S1P, and Cer, collectively constituting the “sphingolipid rheostat” (Figure 2A,B). Particularly noteworthy was the remarkable 60–70-fold increase in S1P levels, accompanied by a 1.5-fold increase in other constituents of the “sphingolipid rheostat.” Intriguingly, complex sphingolipids including HexCer and SM remained unaffected (Figure 2B, Supplementary Figure S2). It is noteworthy that the impaired sphingolipid metabolism due to SPL deficiency did not exhibit a specific preference for ceramide types; for both ceramides (id17:1-Cer) and dihydroCer (id17:0-Cer), ceramides with varying *N*-acyl chain C16~C27 lengths displayed relatively uniform accumulation levels (Figure 2C,D).

### 3.3. Spl-1 RNAi Disrupts Muscle Fiber Organization and Elevates ROS Levels in C. elegans

**Figure 3:**
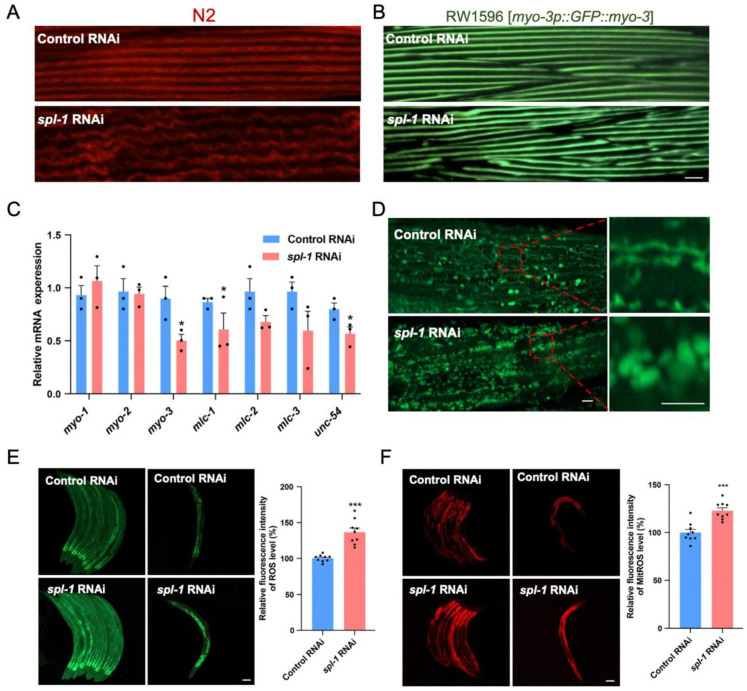
**Functional loss of SPL leads to disrupted myofibril structure and elevated ROS levels**. (**A**) L4-stage wildtype (N2) nematodes were treated with either control or *spl-1* RNAi, and muscle structures were visualized using phalloidin staining after three days. (**B**) L4-stage RW1596 nematodes were subjected to control or *spl-1* RNAi for three days, and the morphology of muscle fibers was assessed. Scale bar, 10 μm. (**C**) The relative mRNA expressions of myosin-related genes *(myo-1/2/3*, *mlc-1/2/3,* and *unc-54*) were analyzed by qRT-PCR. Error bars represent SD (*n* = 3, two-tailed Student’s *t* test, * *p* < 0.05). (**D**) Comparison of body wall muscle mitochondrial morphological between Control RNAi-treated and *spl-1* RNAi-treated SJ4103 strain worms after 3 days under 20 °C, using a mitochondrially localized GFP reporter. The red box represents the area of partial enlargement. Scale bar, 5 μm. (**E**) The reactive oxygen species (ROS) levels of nematodes were assessed by monitoring green fluorescence using dichlorofluorescein (DCF). Scale bar, 100 μm. The rightmost graphs display the quantitative results for each condition. Error bars represent SD (*n* = 3, total 9 worms for each group, two-tailed Student’s *t* test, t = 6.29, *** *p* < 0.001). (**F**) The mitochondria reactive oxygen species (MitoROS) levels of nematodes were assessed by MitoSOX Red. Scale bar, 100 μm. The rightmost graphs display the quantitative results for each condition. Error bars represent SD (*n* = 3, total 9 worms for each group, two-tailed Student’s *t* test, t = 5.57, *** *p* < 0.001).

The accumulation of ceramides has been associated with reduced muscle strength, impaired mobility, and muscle atrophy [24]. To further explore the mechanism underlying decreased mobility in *C. elegans*, we investigated the impact of *spl-1* knockdown on the muscle sarcomere structure. We utilized phalloidin, a dye specific to actin filaments in muscle sarcomeres, to stain nematodes treated with *spl-1* interference bacteria for three days after the L4 stage. Our analysis revealed a significant disruption in muscle sarcomere protein arrangement in *spl-1-*interfered N2 nematodes (Figure 3A). Furthermore, we utilized RW1596 nematodes expressing green fluorescent protein (GFP)-tagged myosin heavy chain (MHC) in body wall muscle cells, which were subjected to *spl-1* RNAi treatment after the L4 stage. Notably, a pronounced morphological disruption in muscle sarcomeres was observed in RW1596 nematodes treated with the *spl-1* interference bacteria compared with control interference nematodes, further confirming the deleterious effect of *spl-1* gene knockdown on muscle fiber structure (Figure 3B). Muscle contraction relies on the interaction between actin and myosin, powered by ATP. Myosin light chains regulate this process. We observed a significant reduction in the mRNA expression levels of *myo-3* (a Myo family gene encoding myosin heavy chain), *mlc-2* (encoding myosin light chain of the MLC family), and *unc-54* (encoding myosin light chain–heavy chain interaction protein) in nematodes treated with *spl-1* interference bacteria compared with control interference nematodes (Figure 3C). 

Mitochondria play a crucial role in ATP production, significantly impacting muscle function. Using SJ4103 nematodes expressing GFP in muscle cells, we observed that *spl-1* RNAi resulted in abnormal enlarged spherical muscle mitochondria, deviating from their original linear network morphology (Figure 3D). Gene expressions associated with mitochondrial fission (*fis-1*, *fis-2*, *mff-1*) and fusion (*fzo-1*, *eat-3*) were suppressed following *spl-1* RNAi (Supplementary Figure S3A). Mitochondrial dysfunction is a pivotal event in cellular homeostasis imbalance, leading to a notable increase in reactive oxygen species (ROS). To investigate whether mitochondrial dysfunction led to subsequent ROS accumulation, we utilized the fluorescent probe DCFH-DA. We observed a significant increase in the average fluorescence intensity of DCFH-DA staining in nematodes treated with *spl-1* RNAi, showing a notable elevation in ROS levels following SPL knockdown (Figure 3E). The mitochondrial ROS accumulation was also evidenced by MitoSOX staining (Figure 3F), thereby indicating mitochondrial dysfunction and oxidative stress resulting from ceramide accumulation.

### 3.4. NAC Supplementation Alleviates Muscle Morphology Defects and Motor Impairment in SPL-Deficient C. elegans

**Figure 4:**
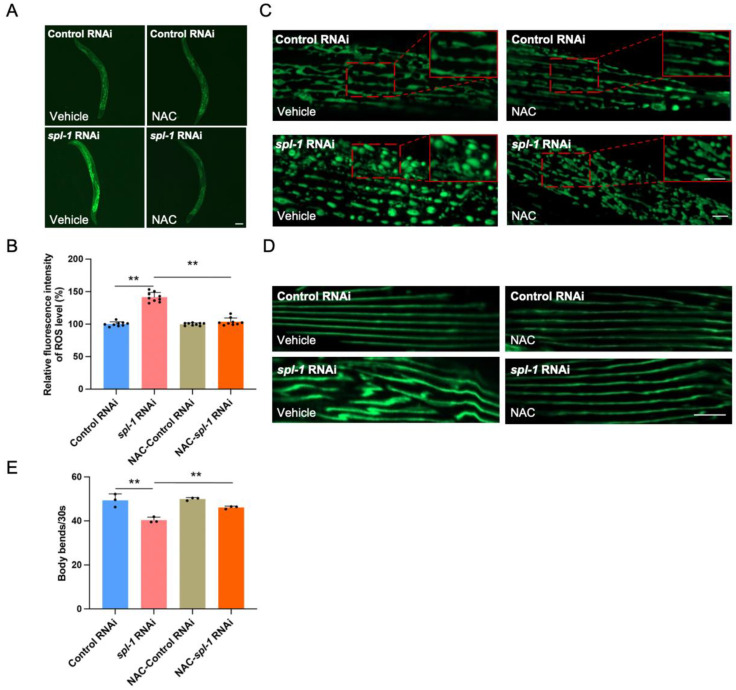
**Supplementation of *N*-acetylcysteine (NAC) inhibits ROS accumulation and rescues myofiber disorganization and motility impairments caused by SPL dysfunction**. (**A**) Three days of 10 mM NAC supplementation to L4-stage nematodes suppressed the ROS accumulation induced by *spl-1* RNAi. Scale bar, 100 μm. (**B**) The graph displays the quantitative results for each condition. Error bars represent SD (*n* = 3, total 9 worms for each group, one-way ANOVA, F = 143.00, ** *p* < 0.01). (**C**) Three days of 10 mM NAC supplementation to L4-stage nematodes can slightly alleviate the damage of mitochondrial morphology caused by *spl-1* RNAi. Scale bar, 5 μm. (**D**) The disorganization of muscle fibers. Scale bar, 100 μm. (**E**) The motility measured as body bends per 30 s were rescued; *n* = 3 for each group; each independent repeat experiment detected 10 worms. The *p* values are relative to *spl-1* RNAi without NAC supplementary animals analyzed by one-way ANOVA, F = 20.48, ** *p* < 0.01.

Due to ceramide-accumulation-induced ROS buildup, SPL deficiency in worms led to a significant elevation in ROS levels. To mitigate this ROS accumulation, we attempted to utilize *N*-acetylcysteine (NAC), a ROS scavenger. NAC serves as a precursor for cysteine and glutathione (GSH) biosynthesis, participating in cellular antioxidant responses. Additionally, NAC directly reacts with ROS, protecting cells from oxidative damage [25]. Using the fluorescent probe DCFH-DA to indicate ROS levels, we found that 10 mM NAC supplementation did not affect the ROS levels in the Control RNAi-treated worms, whereas it effectively cleared the elevated ROS induced by *spl-1* RNAi (Figure 4A,B). While NAC failed to improve the abnormal mitochondrial morphology caused by SPL deficiency (Figure 4C), it also did not influence the gene expressions associated with mitochondrial fission and fusion (Supplementary Figure S3B). Interestingly, in the Control RNAi-treated group, NAC did not affect the morphological characteristics of muscle fibers. NAC supplementation significantly rescued the disorganized muscle fiber phenotype in *spl-1* RNAi-treated nematodes (Figure 4D). Moreover, the motor dysfunction phenotype induced by SPL deficiency was significantly rescued by NAC treatment (Figure 4E). These findings suggest that NAC can restore normal muscle fiber arrangement and rescue motor dysfunction caused by *spl-1* deficiency by clearing the ROS accumulation resulting from ceramide buildup due to the blockage of sphingolipid metabolism.

## 4. Discussion

In this study, we elucidate the critical role of sphingosine phosphate lyase (SPL) in maintaining muscle function in *Caenorhabditis elegans*. By suppressing SPL through *spl-1* RNA interference, we observed decreased motility and muscle fiber disorganization, which were associated with the impaired metabolism of ceramide and mitochondrial dysfunction induced by the accumulation of reactive oxygen species (ROS). Notably, antioxidant *N*-acetylcysteine (NAC) treatment mitigated locomotive capabilities and muscle fiber organization, highlighting its therapeutic potential for ceramide-accumulation-related muscle disorders. This work underscores the importance of SPL in maintaining muscle health and suggests that antioxidants like NAC could be new avenues for corresponding therapeutic intervention.

Sphingolipids, a class of lipids with significant roles in cell membrane function and cell signaling, are subject to stringent metabolic regulation. In skeletal muscle, these lipids play a key role in modulating various signaling pathways within muscle cells, such as STAT3 inflammatory signaling, TGF*β* signaling, calcium mobilization, cell cycle regulation, migration, and fusion [26,27,28,29,30]. The enzyme SPL, critical in sphingolipid metabolism, is essential for maintaining normal functionality. Downregulation of *spl-1* in *C. elegans* results in a shortened lifespan, decreased body movement, and overall motility, similar to the phenotypes observed in *Sgpl-1*-knockout mice [6,15] and SPL-deficient flies [11]. SPL catalyzes the irreversible cleavage of sphingosine-1-phosphate (S1P), representing the only known metabolic exit point for the sphingolipid family. SPL deficiency may impede S1P metabolism, affecting the entire sphingolipid profile and resulting in ceramide accumulation by the salvage pathway [31]. Our findings of increased ceramide levels, particularly C16/C24 ceramides, in SPL-deficient worms are consistent with previous studies in mice [15] and human muscle tissue [20]. Ceramide metabolism homeostasis regulates mitochondrial and protein homeostasis during muscle aging. The accumulation of ceramides in aged muscle contributes to protein aggregation in muscle cells, and inhibiting *de novo* ceramide synthesis can improve muscle quality in aged muscle [21]. Specifically, C18 ceramide, synthesized by ceramide synthase 1 (CerS1), is abundant in skeletal muscle and is suggested to promote insulin resistance in humans [32]. Excessive ceramide accumulation in aged muscle contributes to protein aggregation and mitochondrial dysfunction. In skeletal muscle, the mitochondria form an interconnected network, typically organized in a reticular structure [33]. Maintaining intact mitochondrial morphology is essential for muscle health. CerS6-derived C16:0 ceramides bind to the mitochondrial fission factor, leading to mitochondrial disorders in obese mice [34]. Additionally, ceramides can increase the phosphorylation level of the mitochondrial fission protein DRP1, promoting mitochondrial fission [35]. Our recent research has revealed that insufficient synthesis of key ceramides catalyzed by *hyl-2* dysfunction could also lead to abnormalities in mitochondrial function and morphology [23]. Mitochondrial MFN1 and MFN2 were reported to be suppressed following SPL knockdown in HeLa cells [36]. In SPL-deficient *C. elegans*, we observed suppression of mitochondrial fission- and fusion-related gene expressions, along with disrupted mitochondrial morphology. These mitochondrial disorders may be caused by the combined effects of S1P and ceramide, requiring further investigation.

Disruption of the mitochondrial morphology, leading to the accumulation of reactive oxygen species (ROS), can impair muscle cell structure and function, thereby affecting motility. C16 ceramide inhibits complex IV of the electron transport chain, causing ROS accumulation [37]. SPL deficiency raises ROS and C16 ceramide levels in *C. elegans*, likely by damaging the mitochondrial electron transport chain, thus boosting ROS production. *N-*acetylcysteine (NAC), an intracellular antioxidant, scavenges ROS, protecting against oxidative damage. Studies have shown that NAC can alleviate skeletal muscle inflammation and improve muscle mass in patients with Duchenne muscular dystrophy [38]. In this study, NAC successfully restored motility and muscle fiber organization in SPL-deficient worms but did not improve mitochondrial morphology or the imbalance of mitochondrial dynamics. This conveniently illustrates that mitochondrial damage serves as an upstream trigger for ROS generation. However, this does not exclude the potential benefits of NAC on other aspects of mitochondrial function in SPL-deficient worms. Given that our current experiments are limited to the individual level of *C. elegans* and that we have not yet conducted quantitative analysis of sphingolipids within muscle tissue in the SPL-deficient model, the future work involving the construction of muscle cell and skeleton muscle-targeted SPL-deficient animal models will enable a more comprehensive understanding of the impact of SPL deficiency on sphingolipid metabolism and muscle function. 

## 5. Conclusions

Collectively, we targeted SPL to disrupt sphingolipid metabolism in *C. elegans*, establishing an accumulation model with motor dysfunction. This approach provides a model for investigating interventions for sphingolipid-metabolism-related disorders. Moreover, we revealed the pivotal role of SPL in preserving muscle integrity and advocated exploring antioxidant interventions, such as NAC supplementation, as prospective therapeutic strategies for addressing muscle function decline associated with ceramide accumulation. We believe further experiments in mammals are needed to corroborate the impact on muscle function by sphingolipid metabolism, which could greatly contribute to human health.

## Data Availability

The original contributions presented in the study are included in the article/supplementary material, further inquiries can be directed to the corresponding author due to privacy reasons.

## Supplementary Materials

The following supporting information can be downloaded at: https://www.mdpi.com/article/10.3390/nu16111623/s1, Figure S1. **The *spl-1* RNA interference led to a reduction in brood size but did not affect pharyngeal pumps in *Caenorhabditis elegans***. A. Total brood size comparison between Control RNAi and spl-1 RNAi, analyzed by a two-tailed Student’s *t*-test (*n* = 10, ** *p* < 0.01). B. Pharyngeal pumps were counted for 30 sec for each worm, analyzed by a two-tailed Student’s *t*-test, *n* = 3 for each group, each independent repeat experiment detected 10 worms. Figure S2. **Sphingolipid profiles show no impact on SM or HexCer in SPL loss-of-function *C. elegans***. The heatmaps display the relative changes in the log2 fold change of the C16~C27 *N*-acyl chain lengths of SM (id17:1-SM, A and id17:0-SM, B) and HexCer (id17:1-HexCer, C and id17:0-Hex, D) in nematodes, generated using the R pheatmap package. Figure S3. **Supplementation of *N*-acetylcysteine (NAC) failed to improve the mitochondrial fission and fusion abnormalities caused by SPL functional loss**. A. Quantitative RT-PCR analyses were conducted on the expression of 5 mitochondrial fission genes and 2 fusion genes between Control RNAi-treated and *spl-1* RNAi-treated N2 worms after 3 days under 20 °C. All *p*-values were calculated by two-tailed Student’s *t*-test (* *p* < 0.05; ** *p* < 0.01). B. NAC supplementation failed to rescue the mRNA expression of mitochondrial fission and fusion genes in *spl-1* RNAi. Table S1. Primers used in this study. Table S2. MRM pair of sphingolipids analysis by LC-MS/MS. Table S3. LC condition of sphingolipids analysis.
