# Supplementary material for: N-Acetylcysteine Alleviates Impaired Muscular Function Resulting from Sphingosine Phosphate Lyase Functional Deficiency-Induced Sphingoid Base and Ceramide Accumulation in Caenorhabditis elegans
Source: Nutrients. 2024 May 26;16(11):1623. doi: 10.3390/nu16111623 (PMC11174433; doi:10.3390/nu16111623)
Supplement: Supplementary file 1 [file nutrients-16-01623-s001.zip › nutrients-2991506-supplementary.pdf]

N-Acetylcysteine Alleviates Impaired Muscular Function Resulting from Sphingosine Phosphate Lyase Functional Deficiency-Induced Sphingoid Base and Ceramide Accumulation in *Caenorhabditis elegans*

Min Liu [†], Yunfei You[†], Huaiyi Zhu[†], Yu Chen, Zhenying Hu*, and Jingjing Duan*

Jiangxi Province Key Laboratory of Aging and Disease, Human Aging Research Institute (HARI), School of Life Science, Nanchang University, Nanchang 330031, China; liumin_w@163.com (M.L.); youyunfei1027@163.com (Y.Y.); huaiyi_zhu@email.ncu.edu.cn (H.Z.); ncuskchenyu2020@163.com (Y.C.)

* Correspondence: whozing@aliyun.com (Z.H.); duan.jingjing@ncu.edu.cn (J.D.)

[†] These authors contributed equally to this work.

Supplementary Materials:

Figure S1. The *spl-1* RNA interference led to a reduction in brood size but did not affect pharyngeal pumps in *Caenorhabditis elegans.* A. Total brood size comparison between Control RNAi and *spl-1* RNAi, analyzed by a two-tailed Student's *t* test (n=10, **$p < 0.01$). B. Pharyngeal pumps were counted for 30 sec for each worm, analyzed by a two-tailed Student's *t* test, $n = 3$ for each group, each independent repeat experiment detected 10 worms.

Figure S2. Sphingolipid profiles show no impact on SM or HexCer in SPL loss-of -function *C. elegans.* The heatmaps display the relative changes in the log2 fold change of the C16~C27 *N*-acyl chain lengths of SM (id17:1-SM, A and id17:0-SM, B) and HexCer (id17:1-HexCer, C and id17:0-Hex, D) in nematodes, generated using the R pheatmap package.

Figure S3. Supplementation of *N*-acetylcysteine (NAC) failed to improve the mitochondrial fission and fusion abnormalities caused by SPL functional loss. A. Quantitative RT-PCR analyses were conducted on the expression of 5 mitochondrial fission genes and 2 fusion genes between Control RNAi-treated and *spl-1* RNAi-treated N2 worms after 3 days under 20°C. All *P*-values were calculated by two-tailed Student's t test (*$p < 0.05$; **$p < 0.01$). B. NAC supplementation failed to rescue the mRNA expression of mitochondrial fission and fusion genes in *spl-1* RNAi.

Table S1 Primers used in this study

Gene	Primers (5’→3’)
spl-1	F: AGCGCCTAACTTCCCATCAG<br>R: TTCGGTGTGCATCCGTACTT
fzo-1	F: ACCGGTGGCCTAATGTCAAG<br>R: GAGCAGGGACATCGACACAA
eat-3	F: GAGAACTGGAACGCCTCGAA<br>R: ACTACTACAACACGCGGCAA
drp-1	F: GAGAAAGAGCGGTCAGTGCT<br>R: ACGTTGGTTCTGGGATTGCT
mff-1	F: TGCAAAACCCGTACGAGGAA<br>R: CGTCGGAGGTATGAGTATGCC
mff-2	F: ATCCGGTTGAAGATCAGCGG<br>R: TGGCTTGCTCTCGATGTCTG
fis-1	F: CTCTTCTCCGAACCGAACCA<br>R: CGAGCAATCCAAGTCCCAGA
fis-2	F: ACGATGAGGACAGAACGAGC<br>R: TCCTTCAACGTCTTCGCCTG
mlc-1	F: GCCAGATCGACGCCATGATTAAA<br>R: ATAATGGTGGCCTCTGGGTCA
mlc-2	F: GGGAGAGATTGACAGGAACTGAC<br>R: CCTCTCCTCCCTCGATTGGT
mlc-3	F: CGGAACCCAAGTCGGAGATG<br>R: AGTCAGCGTAGGTTCCTTGC
unc-54	F: GAGCGCTCCAAGAAGGCTAT<br>R: GAGCTGTTGCTCAAGTCCCT
myo-1	F: GAACACGAGAAGGACCCAGG<br>R: TCTTCGAATCGTATGGGCGG
myo-2	F: AGCATCCAAGACCCTTGCTC<br>R: GGCTGTTAGCTTGCTCCTCA
myo-3	F: ACAGACTGCAGGAGGAAAGC<br>R: GGGGAATCCCTTACGGCAAA

Table S2 MRM pair of sphingolipids analysis by LC-MS/MS

Method #1

Q1	Q3	R.T. (min)	ID	DP	CE
286.3	268.3	3.12 (id17:1), 3.24 (d17:1, IS*)	d17:1	70	20
288.3	270.3	3.45 (id17:0), 3.56 (d17:0, IS)	d17:0	110	20
366.3	250.3	3.34 (id17:1-S1P), 3.46 (d17:1-S1P, IS)	d17:1-S1P	70	20
368.3	270.3	3.67 (id17:0-S1P), 3.79 (d17:0-S1P)	d17:0-S1P	100	20
268.3	250.3	3.1	m17:1	70	20
270.3	252.3	3.38	m17:0	80	25

*IS: internal standard

Method #2

Q1	Q3	R.T. (min)	ID	DP	CE	Q1	Q3	R.T. (min)	ID	DP	CE
510.5	254.3	4.43	m17:0/16:0	100	35	810.7	250.3	6.4	HexCer-d17:1/25:1	70	45
538.5	254.3	5.21	m17:0/18:0	100	35	826.7	250.3	6.65	HexCer-d17:1/26:0	70	45
554.6	254.3	4.3	m17:0/18:0h	100	35	842.7	250.3	6.8	HexCer-d17:1/26:0h	70	45
536.5	254.3	4.39	m17:0/18:1	100	35	824.7	250.3	6.7	HexCer-d17:1/26:1	70	45
550.6	254.3	5.44	m17:0/19:1	100	35	840.7	250.3	6.1	HexCer-d17:1/26:1h	70	45
610.6	254.3	5.76	m17:0/22:0h	100	35	840.7	250.3	6.9	HexCer-d17:1/27:0	70	45
592.6	254.3	5.9	m17:0/22:1	100	35	856.7	250.3	6.9	HexCer-d17:1/27:0h	70	45
524.5	250.3	4.27	d17:1/16:0	100	35	838.7	250.3	7	HexCer-d17:1/27:1	70	45
540.5	250.3	3.9	d17:1/16:0h	100	35	700.6	252.3	4	HexCer-d17:0/17:1	70	45
522.5	250.3	3.91	d17:1/16:1	100	35	774.6	252.3	5.14	HexCer-d17:0/21:0h	70	45
538.5	250.3	4.55	d17:1/17:0	100	35	756.6	252.3	5.12	HexCer-d17:0/21:1	70	45
554.5	250.3	4.17	d17:1/17:0h	100	35	772.6	252.3	5.5	HexCer-d17:0/21:1h	70	45

536.5	250.3	4.14	d17:1/17:1	100	35	772.6	252.3	5.45	HexCer-d17:0/22:0	70	45
552.5	250.3	5.04	d17:1/18:0	100	35	788.7	252.3	5.5	HexCer-d17:0/22:0h	70	45
568.5	250.3	4.65	d17:1/18:0h	100	35	770.7	252.3	5.47	HexCer-d17:0/22:1	70	45
550.5	250.3	4.65	d17:1/18:1	100	35	786.6	252.3	5.44	HexCer-d17:0/22:1h	70	45
566.5	250.3	5.32	d17:1/19:0	100	35	786.6	252.3	5.5	HexCer-d17:0/23:0	70	45
580.5	250.3	5.78	d17:1/20:0	100	35	802.7	252.3	5.8	HexCer-d17:0/23:0h	70	45
596.6	250.3	5.41	d17:1/20:0h	100	35	784.7	252.3	5.8	HexCer-d17:0/23:1	70	45
578.6	250.3	5.4	d17:1/20:1	100	35	800.7	252.3	5.7	HexCer-d17:0/23:1h	70	45
594.5	250.3	6.14	d17:1/21:0	100	35	800.7	252.3	6.1	HexCer-d17:0/24:0	70	45
610.6	250.3	5.76	d17:1/21:0h	100	35	816.7	252.3	6.1	HexCer-d17:0/24:0h	70	45
592.6	250.3	5.77	d17:1/21:1	100	35	798.7	252.3	6.1	HexCer-d17:0/24:1	70	45
608.6	250.3	6.49	d17:1/22:0	100	35	814.7	252.3	6.1	HexCer-d17:0/24:1h	70	45
624.6	250.3	6.11	d17:1/22:0h	100	35	814.7	252.3	6.5	HexCer-d17:0/25:0	70	45
606.6	250.3	6.11	d17:1/22:1	100	35	830.7	252.3	6.4	HexCer-d17:0/25:0h	70	45
622.6	250.3	5.8	d17:1/22:1h	100	35	812.7	252.3	6.4	HexCer-d17:0/25:1	70	45
622.6	250.3	6.84	d17:1/23:0	100	35	828.7	252.3	6.4	HexCer-d17:0/25:1h	70	45
638.6	250.3	6.45	d17:1/23:0h	100	35	828.7	252.3	6.6	HexCer-d17:0/26:0	70	45
620.6	250.3	6.45	d17:1/23:1	100	35	844.7	252.3	6.6	HexCer-d17:0/26:0h	70	45
636.6	250.3	6.7	d17:1/23:1h	100	35	826.7	252.3	6.7	HexCer-d17:0/26:1	70	45
636.6	250.3	7.16	d17:1/24:0	100	35	842.7	252.3	6.7	HexCer-d17:0/26:1h	70	45
652.6	250.3	6.79	d17:1/24:0h	100	35	858.7	252.3	6.7	GluCer-d17:0/27:0h	70	45
634.6	250.3	6.8	d17:1/24:1	100	35	840.7	252.3	6.9	GluCer-d17:0/27:1	70	45
650.6	250.3	7.1	d17:1/24:1h	100	35	691.5	184.3	3.51	SM-d17:0/16:0	100	35
650.6	250.3	7.4	d17:1/25:0	100	35	707.6	184.3	3.08	SM-d17:0/16:0h	100	35
666.6	250.3	7.02	d17:1/25:0h	100	35	705.6	184.3	3.75	SM-d17:0/17:0	100	35

648.6	250.3	7.06	d17:1/25:1	100	35	719.6	184.3	3.99	SM-d17:0/18:0	100	35
664.6	250.3	7.4	d17:1/25:1h	100	35	735.6	184.3	3.8	SM-d17:0/18:0h	100	35
664.6	250.3	7.71	d17:1/26:0	100	35	733.6	184.3	4.2	SM-d17:0/19:0	100	35
680.7	250.3	7.41	d17:1/26:0h	100	35	747.6	184.3	4.9	SM-d17:0/20:0	100	35
662.6	250.3	7.4	d17:1/26:1	100	35	745.6	184.3	4.02	SM-d17:0/20:1	100	35
678.6	250.3	7.91	d17:1/27:0	100	35	761.6	184.3	5.21	SM-d17:0/21:0	100	35
694.7	250.3	7.61	d17:1/27:0h	100	35	777.6	184.3	4.99	SM-d17:0/21:0h	100	35
676.7	250.3	7.61	d17:1/27:1	100	35	759.6	184.3	4.37	SM-d17:0/21:1	100	35
708.7	250.3	7.8	d17:1/28:0h	100	35	775.6	184.3	5.5	SM-d17:0/22:0	100	35
690.7	250.3	7.8	d17:1/28:1	100	35	791.7	184.3	5.36	SM-d17:0/22:0h	100	35
526.5	252.3	4.55	d17:0/16:0	100	35	773.6	184.3	5.4	SM-d17:0/22:1	100	35
540.5	252.3	4.6	d17:0/17:0	100	35	789.6	184.3	5.39	SM-d17:0/22:1h	100	35
538.5	252.3	4.75	d17:0/17:1	100	35	789.6	184.3	5.9	SM-d17:0/23:0	100	35
554.5	252.3	5.05	d17:0/18:0	100	35	805.7	184.3	5.3	SM-d17:0/23:0h	100	35
598.6	252.3	5.4	d17:0/20:0h	100	35	787.6	184.3	5.3	SM-d17:0/23:1	100	35
580.6	252.3	5.4	d17:0/20:1	100	35	803.7	184.3	6.29	SM-d17:0/24:0	100	35
596.6	252.3	6.11	d17:0/21:0	100	35	819.7	184.3	6	SM-d17:0/24:0h	100	35
612.6	252.3	5.84	d17:0/21:0h	100	35	801.6	184.3	6	SM-d17:0/24:1	100	35
594.6	252.3	5.8	d17:0/21:1	100	35	817.7	184.3	5.8	SM-d17:0/24:1h	100	35
610.6	252.3	6.71	d17:0/22:0	100	35	817.7	184.3	6.5	SM-d17:0/25:0	100	35
626.6	252.3	6.1	d17:0/22:0h	100	35	833.7	184.3	6.22	SM-d17:0/25:0h	100	35
608.6	252.3	6.11	d17:0/22:1	100	35	831.7	184.3	6.4	SM-d17:0/26:0	100	35
624.6	252.3	5.52	d17:0/22:1h	100	35	847.7	184.3	6.3	SM-d17:0/26:0h	100	35
624.6	252.3	6.79	d17:0/23:0	100	35	845.7	184.3	6.9	SM-d17:0/27:0	100	35
640.6	252.3	6.5	d17:0/23:0h	100	35	861.7	184.3	6.6	SM-d17:0/27:0h	100	35

622.6	252.3	6.5	d17:0/23:1	100	35	843.7	184.3	6.6	SM-d17:0/27:1	100	35
638.6	252.3	7.3	d17:0/24:0	100	35	875.8	184.3	6.6	SM-d17:0/28:0h	100	35
654.6	252.3	6.75	d17:0/24:0h	100	35	689.5	184.3	3.5	SM-d17:1/16:0	100	35
636.6	252.3	6.79	d17:0/24:1	100	35	705.6	184.3	3.15	SM-d17:1/16:0h	100	35
652.6	252.3	6.1	d17:0/24:1h	100	35	687.5	184.3	3	SM-d17:1/16:1	100	35
652.6	252.3	7.52	d17:0/25:0	100	35	703.5	184.3	3.75	SM-d17:1/17:0	100	35
668.7	252.3	7.08	d17:0/25:0h	100	35	719.6	184.3	3.69	SM-d17:1/17:0h	100	35
650.6	252.3	7.12	d17:0/25:1	100	35	701.6	184.3	3.35	SM-d17:1/17:1	100	35
666.6	252.3	7.85	d17:0/26:0	100	35	717.6	184.3	4.23	SM-d17:1/18:0	100	35
682.7	252.3	7.49	d17:0/26:0h	100	35	733.6	184.3	4.2	SM-d17:1/18:0h	100	35
664.7	252.3	7.41	d17:0/26:1	100	35	715.6	184.3	3.57	SM-d17:1/18:1	100	35
680.7	252.3	7.9	d17:0/27:0	100	35	731.6	184.3	3.9	SM-d17:1/19:0	100	35
696.7	252.3	7.9	d17:0/27:0h	100	35	745.6	184.3	5.02	SM-d17:1/20:0	100	35
678.7	252.3	7.7	d17:0/27:1	100	35	761.6	184.3	4.95	SM-d17:1/20:0h	100	35
702.6	250.3	3.32	HexCer-d17:1/16:0h	70	45	743.6	184.3	4.19	SM-d17:1/20:1	100	35
684.5	250.3	3.31	HexCer-d17:1/16:1	70	45	759.6	184.3	5.42	SM-d17:1/21:0	100	35
716.6	250.3	3.58	HexCer-d17:1/17:0h	70	45	775.6	184.3	5.01	SM-d17:1/21:0h	100	35
698.6	250.3	3.57	HexCer-d17:1/17:1	70	45	773.6	184.3	5.79	SM-d17:1/22:0	100	35
730.6	250.3	4.01	HexCer-d17:1/18:0h	70	45	789.6	184.3	5.38	SM-d17:1/22:0h	100	35
712.6	250.3	4.03	HexCer-d17:1/18:1	70	45	771.6	184.3	5.38	SM-d17:1/22:1	100	35
728.6	250.3	3.4	HexCer-d17:1/18:1h	70	45	787.6	184.3	5	SM-d17:1/22:1h	100	35
758.6	250.3	4.75	HexCer-d17:1/20:0h	70	45	787.6	184.3	6.17	SM-d17:1/23:0	100	35
740.6	250.3	4.76	HexCer-d17:1/20:1	70	45	803.7	184.3	5.78	SM-d17:1/23:0h	100	35
756.6	250.3	5.1	HexCer-d17:1/21:0	70	45	785.7	184.3	5.6	SM-d17:1/23:1	100	35
772.6	250.3	5.1	HexCer-d17:1/21:0h	70	45	801.6	184.3	6.4	SM-d17:1/24:0	100	35

754.6	250.3	5.1	HexCer-d17:1/21:1	70	45	817.7	184.3	6.12	SM-d17:1/24:0h	100	35
770.6	250.3	4.73	HexCer-d17:1/21:1h	70	45	799.7	184.3	6.12	SM-d17:1/24:1	100	35
770.6	250.3	5.56	HexCer-d17:1/22:0	70	45	815.7	184.3	6.6	SM-d17:1/25:0	100	35
786.6	250.3	5.46	HexCer-d17:1/22:0h	70	45	831.7	184.3	6.24	SM-d17:1/25:0h	100	35
768.6	250.3	5.47	HexCer-d17:1/22:1	70	45	813.7	184.3	6.24	SM-d17:1/25:1	100	35
784.6	250.3	4.9	HexCer-d17:1/22:1h	70	45	829.7	184.3	7.2	SM-d17:1/26:0	100	35
784.6	250.3	5.81	HexCer-d17:1/23:0	70	45	845.7	184.3	7	SM-d17:1/26:0h	100	35
800.7	250.3	5.81	HexCer-d17:1/23:0h	70	45	827.7	184.3	7	SM-d17:1/26:1	100	35
782.7	250.3	5.79	HexCer-d17:1/23:1	70	45	843.7	184.3	7.3	SM-d17:1/27:0	100	35
798.6	250.3	6.13	HexCer-d17:1/24:0	70	45	859.7	184.3	7.1	SM-d17:1/27:0h	100	35
814.7	250.3	6.14	HexCer-d17:1/24:0h	70	45	841.7	184.3	7.1	SM-d17:1/27:1	100	35
796.7	250.3	6.15	HexCer-d17:1/24:1	70	45	482.5	264.3	3.3 IS	Cer-d18:1/12:0	100	35
812.7	250.3	5.6	HexCer-d17:1/24:1h	70	45	647.6	184.3	2.6 IS	SM-d18:1/12:0	100	35
812.7	250.3	6.36	HexCer-d17:1/25:0	70	45	644.7	264.3	2.6 IS	GluCer-d18:1/12:0	70	45
828.7	250.3	6.46	HexCer-d17:1/25:0h	70	45						

Table S3 LC condition of sphingolipids analysis

Parameters	Method #1	Method #2
Target	id17:1, id17:0, id17:1-S1P, id17:0-S1P, im17:1, im17:0	id17:1-Cer, id17:0-Cer, id17:1-SM, id17:0-SM, id17:1-HexCer, id17:0-HexCer, im17:0-Cer
Column	2.6 μm, 100 × 2.1 mm, 100Å, Phenomenex	2.6 μm, 100 × 2.1 mm, 100Å, Phenomenex
Mobile phases	A. methanol: water: formic acid=58:41:1<br>5 mM ammonium formate<br>B. methanol: formic acid=99:1<br>5 mM ammonium formate	A. methanol: water: acetonitrile = 1:1:1<br>7 mM ammonium acetate<br>B. isopropanol<br>7 mM ammonium acetate
Elution requirement	0-6 min, 45-100% B<br>6-7.5 min, 100% B<br>7.5-7.6 min, 100-45% B<br>7.6-8 min, 45% B	0-1 min, 50% B<br>1-10.2 min, 50-90% B<br>10.2-12.5 min, 90% B<br>12.5-12.6 min, 90-50% B<br>12.6-14 min, 50% B
Flow rate	0.3 ml/min	0.3 ml/min
Oven temperature	40℃	40℃
Equilibrium time	0.2 min	0.2 min
Loading volume	2 μL	2 μL

S-Fig.S1

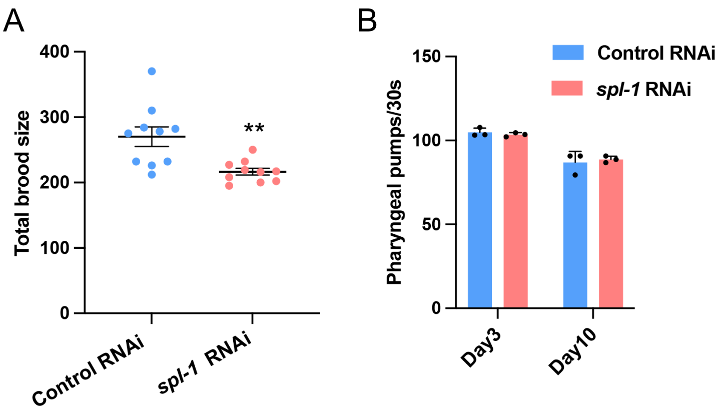

A
Total brood size
400
300
200
100
0
**
Control RNAi
spl-1 RNAi
B
Pharyngeal pumps/30s
150
100
50
0
Control RNAi
spl-1 RNAi
Day3
Day10

S-Fig.S2

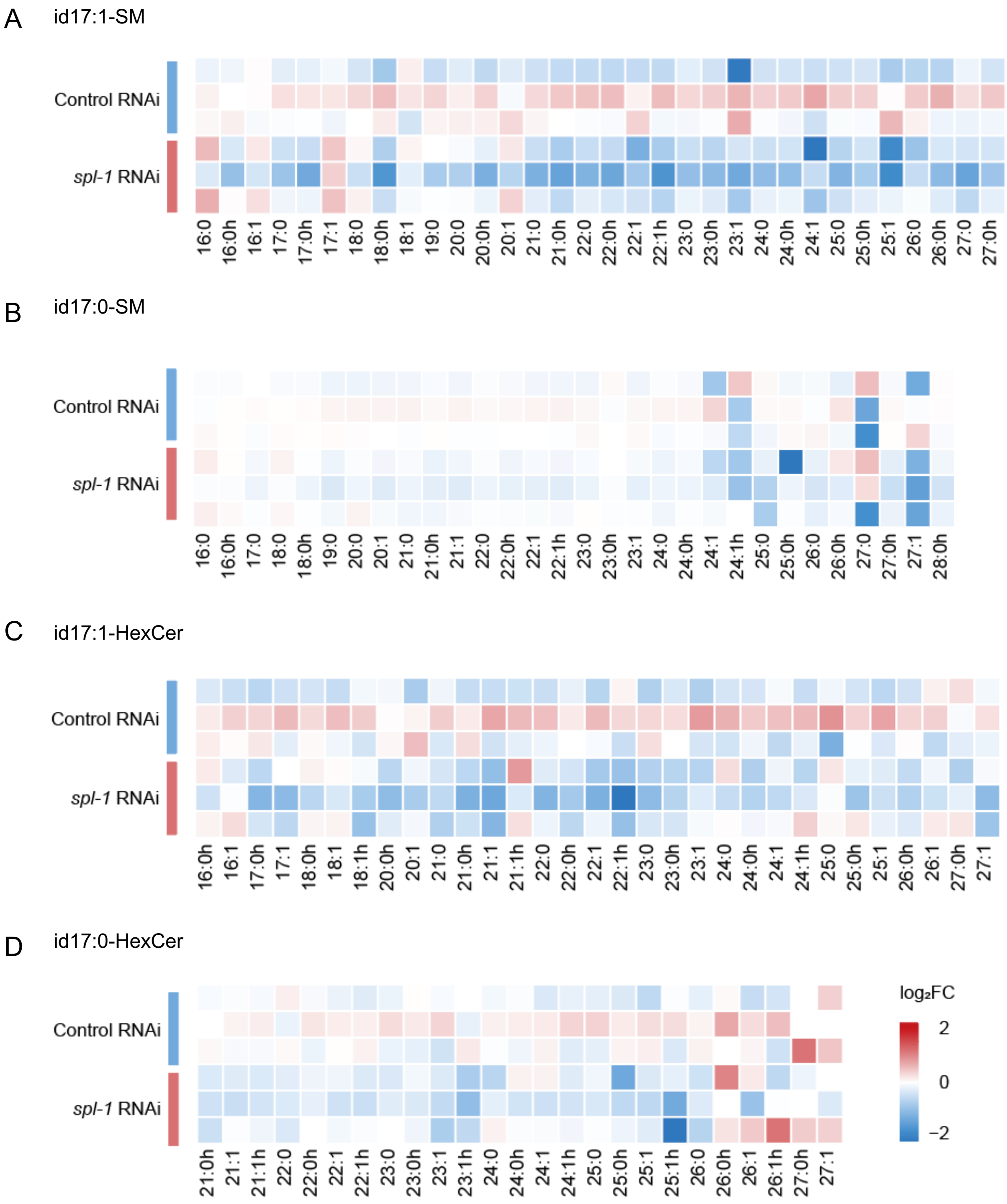
A
id17:1-SM
Control RNAi
spl-1 RNAi
16:0 16:0h 16:1 17:0 17:0h 17:1 18:0 18:0h 18:1 19:0 20:0 20:0h 20:1 21:0 21:0h 22:0 22:0h 22:1 22:1h 23:0 23:0h 23:1 24:0 24:0h 24:1 25:0 25:0h 25:1 26:0 26:0h 27:0 27:0h
B
id17:0-SM
Control RNAi
spl-1 RNAi
16:0 16:0h 17:0 18:0 18:0h 19:0 20:0 20:1 21:0 21:0h 21:1 22:0 22:0h 22:1 22:1h 23:0 23:0h 23:1 24:0 24:0h 24:1 24:1h 25:0 25:0h 26:0 26:0h 27:0 27:0h 27:1 28:0h
C
id17:1-HexCer
Control RNAi
spl-1 RNAi
16:0h 16:1 17:0h 17:1 18:0h 18:1 18:1h 20:0h 20:1 21:0 21:0h 21:1 21:1h 22:0 22:0h 22:1 22:1h 23:0 23:0h 23:1 24:0 24:0h 24:1 24:1h 25:0 25:0h 25:1 26:0h 26:1 27:0h 27:1
D
id17:0-HexCer
Control RNAi
spl-1 RNAi
21:0h 21:1 21:1h 22:0 22:0h 22:1 22:1h 23:0 23:0h 23:1 23:1h 24:0 24:0h 24:1 24:1h 25:0 25:0h 25:1 25:1h 26:0 26:0h 26:1 26:1h 27:0h 27:1
log₂FC
2
0
−2

S-Fig.S3

A

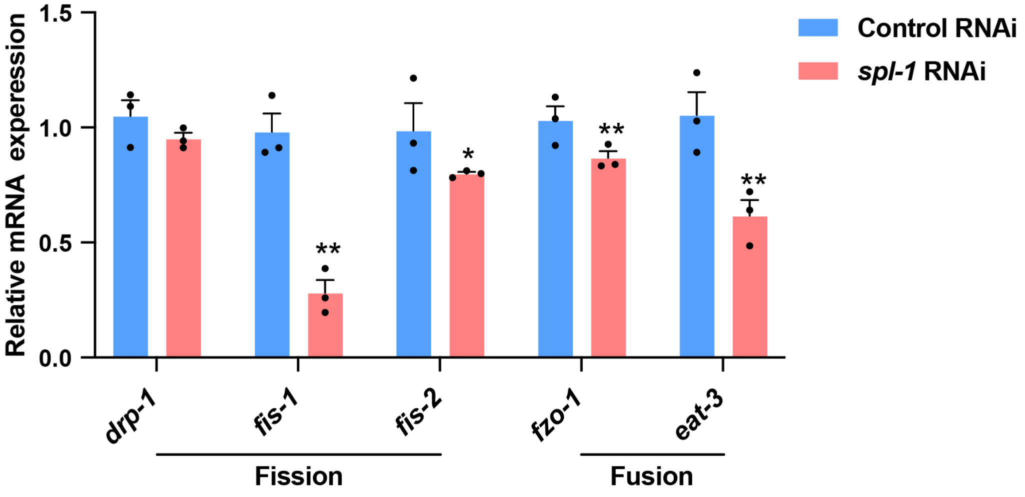

Control RNAi
spl-1 RNAi
Relative mRNA experession
1.5
1.0
0.5
0.0
**
*
**
**
drp-1
fis-1
fis-2
fzo-1
eat-3
Fission
Fusion

B

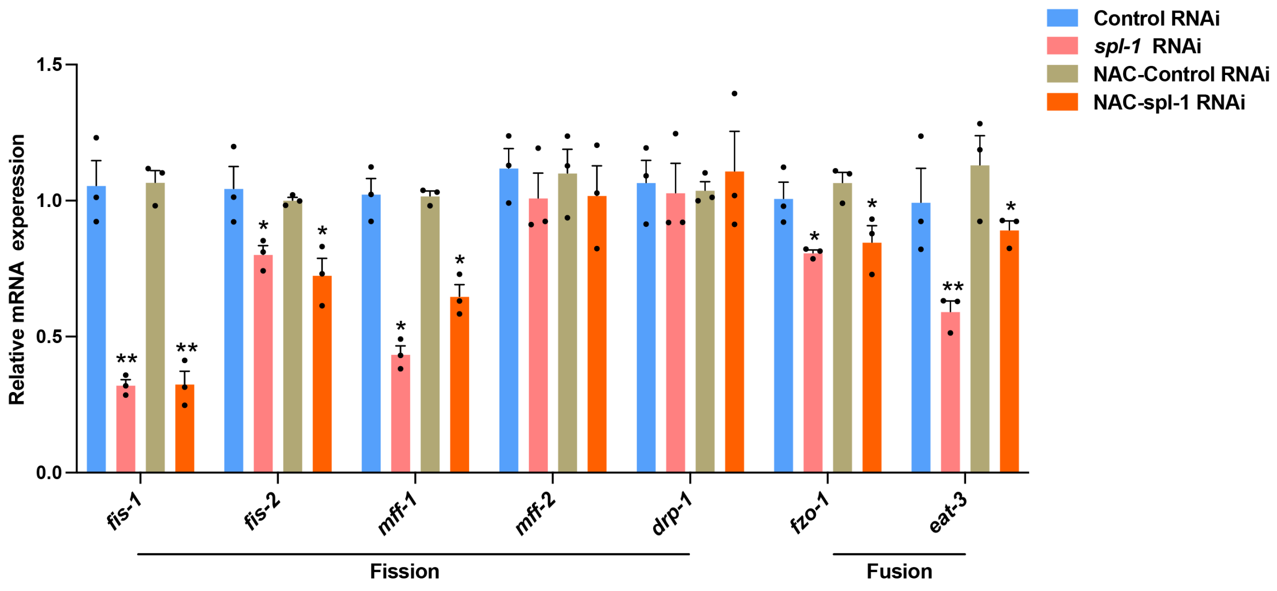

Control RNAi
spl-1 RNAi
NAC-Control RNAi
NAC-spl-1 RNAi
Relative mRNA experession
1.5
1.0
0.5
0.0
fis-1
fis-2
mff-1
mff-2
drp-1
fzo-1
eat-3
Fission
Fusion
